# Nuclear microsatellites reveal population genetic structuring and fine-scale pattern of hybridization in the Japanese mantis shrimp *Oratosquilla oratoria*

**DOI:** 10.7717/peerj.10270

**Published:** 2020-11-05

**Authors:** Jiao Cheng, Nan Zhang, Zhongli Sha

**Affiliations:** 1 Institute of Oceanology, Chinese Academy of Sciences, Qingdao, China; 2 Center for Ocean Mega-Science, Chinese Academy of Sciences, Qingdao, China; 3 Laboratory for Marine Biology and Biotechnology, Qingdao National Laboratory for Marine Science and Technology, Qingdao, China

**Keywords:** Allopatric diversification, Introgressive hybridization, Microsatellites, Marine invertebrates, Mantis shrimp

## Abstract

The interplay between historical and contemporary processes can produce complex patterns of genetic differentiation in the marine realm. Recent mitochondrial and nuclear sequence analyses revealed cryptic speciation in the Japanese mantis shrimp *Oratosquilla oratoria*. Herein, we applied nuclear microsatellite markers to examine patterns and causes of genetic differentiation in this morphotaxon. Population structure analyses revealed two genetically divergent and geographically structured clades in *O. oratoria*, one dominating the temperate zone of the Northwestern (NW) Pacific and the other occurring in the subtropical and tropical waters where are influenced by the Kuroshio Current. Two sympatric zones, one around the Changjiang Estuary in China coast and the other in the northern Japan Sea, were demonstrated to be hybrid zones where introgressive hybridization occurred asymmetrically. The interaction between historical climate shifts and contemporary factors (e.g., freshwater discharge, temperature gradient and isolation by distance) may contribute to the present-day genetic architecture in the Japanese mantis shrimp. Range shift induced by climate changes and oceanographic factors may promote hybridization and gene flow between the *O. oratoria* complex. Our results provide insights into the interacting mechanisms that give rise to diversification and speciation of coastal species in the NW Pacific.

## Introduction

Historic disturbances have been recognized to play a major role in affecting the distribution and abundance, and in shaping the population structure of marine organisms (Grantham, Eckert & Shanks, 2003; Sala-Bozano, Ketmaier & Mariani, 2009; Wilson & Veraguth, 2010). Plio-Pleistocene glacial cycles induced geological and climatic changes in the marginal seas of the Northwestern (NW) Pacific, where several marginal seas, namely the Japan Sea, the South China Sea, and the East China and Yellow Seas, are separated from the open ocean by a series of islands, namely the main Japanese, Ryukyu and Taiwan islands. Sea levels during glacial maxima were approximately 120–140 m lower than present (Wang, 1999). Sea-level fluctuation associated with glacial cycles caused the recurrent closure of the Japan Sea, the semi-closure of the South China Sea and the partial or full exposure of the East China and Yellow Seas (Wang, 1999). In addition to changes in shoreline and sea-basin configuration, the glacial–interglacial cycles also gave rise to pronounced fluctuations in sea temperature, nutrient input and habitat availability (Shen et al., 2011; He et al., 2014). All of these and related environmental factors are believed to have altered geographic ranges and established refugial populations for many coastal marine species (Ni et al., 2014). In the NW Pacific, increasing phylogeographic evidence has emphasized the potential importance of the decreasing sea level and glaciations as drivers of genetic diversity in marine species (e.g., Tzeng, Yeh & Hui, 2004; Liu et al., 2007; Xu et al., 2009; Hu et al., 2015). Climate changes associated with glacial cycles not only promote isolation but also increase the chances of contact and subsequent genetic change between allopatric populations or species as a consequence of repeated range shifts (Mimura et al., 2014). However, the nature of biogeographic responses to glaciation probably varies among taxa. For example, some species likely moved with their optimal habitats, while other species stayed put and adapted to their altered local environments (Liu et al., 2006; Shen et al., 2011; Cheng et al., 2018).

In addition to historical processes, contemporary forces are believed to have an influence on spatial distributions and genetic structuring of marine species, including ocean fronts and currents, environmental gradients in water bodies and intrinsic characteristics of the organisms (Zardoya et al., 2004; Ni et al., 2012; Guo et al., 2015). The adults of many benthic marine invertebrates are sessile or relatively immobile, such that connectivity among populations is mainly ensured by mobility of larvae. Multiple factors are accountable for the frequency of long-distance dispersal, including larval mortality, the effective population size of parental population and the probability of larvae being carried by oceanographic current systems (Scheltema, 1971; Doherty, Planes & Mather, 1995). Panmixia is predicted for species with long planktonic larval stages as these larvae can disperse over large geographical scales when advected by oceanic currents, eventually facilitating gene flow and dispelling genetic differentiation (Arnaud et al., 2000). However, this hypothesis is challenged by recent studies that fine population structure has been increasingly detected in marine invertebrates with extended larval phases (e.g., Luttikhuizen, Drent & Baker, 2003; Zhan et al., 2009; Ni et al., 2012). A weak correlation has been found between pelagic larval duration and population structure (*F*_ST_), likely due to temporal variability, vertical behavior in the open-ocean migration as well as dynamic oceanographic features such as freshwater outflow, mesoscale eddies, upwelling systems and fronts (Weersing & Toonen, 2009; White et al., 2010; Watson et al., 2012).

The Japanese mantis shrimp *Oratosquilla oratoria* De Haan, 1844 is a representative stomatopod crustacean endemic to the NW Pacific coast and is introduced to Australian and New Zealand waters (Ahyong, 2010). It is widely distributed in the NW Pacific from Peter the Great Bay, Russia through the coastal waters of Japan, Korea and China (Komai, 1927; Manning, 1971), and is commercially harvested in Asian countries (Kodama, Shimizu & Aoki, 2003; Yeon & Park, 2011). This species lives in soft sediments, sand and mud in sheltered bays and estuaries, and inhabits U-type burrows up to 30 cm deep (Hamano & Matsuura, 1986). Despite benthic habitat for adults, *O. oratoria* has planktonic larvae and these larvae are known to survive up to 59 days under laboratory conditions (Hamano & Matsuura, 1987), indicating high potential for dispersal during its larval stage. With *O. oratoria* as a model organism, we have previously investigated its phylogeography and demographic history by analyzing mitochondrial cytochrome oxidase subunit I (mtDNA COI) and nuclear ribosomal internal transcribed spacer (nrDNA ITS) sequences (Cheng & Sha, 2017). From sequence analysis, two cryptic species were revealed in *O. oratoria* with clear allopatric distribution. Moreover, we identified two sympatric zones, one in the southern Yellow Sea and the other in the south coast of Japan. This phylogeographic structure may reveal signals of post-glacial secondary contact after an ancient isolation (Cheng & Sha, 2017). Nevertheless, one cannot exclude the relevance of other processes such as incomplete lineage sorting, introgressive hybridization, or local adaptation (Silva et al., 2014; Zhou et al., 2017) in promoting the observed genetic pattern.

With regards to population subdivision of *O. oratoria*, previous findings are somewhat conflicting. Zhang et al. (2014) and Zhang et al. (2016) found a north-south population structure in China Sea as a consequence of long-term isolation by the Taiwan Strait. On the other hand, recent studies reported different pattern of genetic differentiation among China coastal populations, which is in line with the Changjiang Estuary (Du et al., 2016; Cheng & Sha, 2017). Furthermore, previous genetic surveys are mainly based on mitochondrial markers, which make it difficult to identify factors responsible for microevolutionary processes over contemporary timescales and to characterize hybridization. In this study, we apply nuclear microsatellites to (i) clarify population genetic structure of *O. oratoria* over most of its geographical range in the NW Pacific by using population genetic approaches; (ii) depict hybridization in the sympatric areas by studying the ancestry and interspecific heterozygosity of hybrids. Based on these analyses, we further discuss the potential factors driving complex genetic patterns and fine-scale admixture in the Japanese mantis shrimp.

## Materials & Methods

### Sample collection and DNA extraction

**Figure 1:**
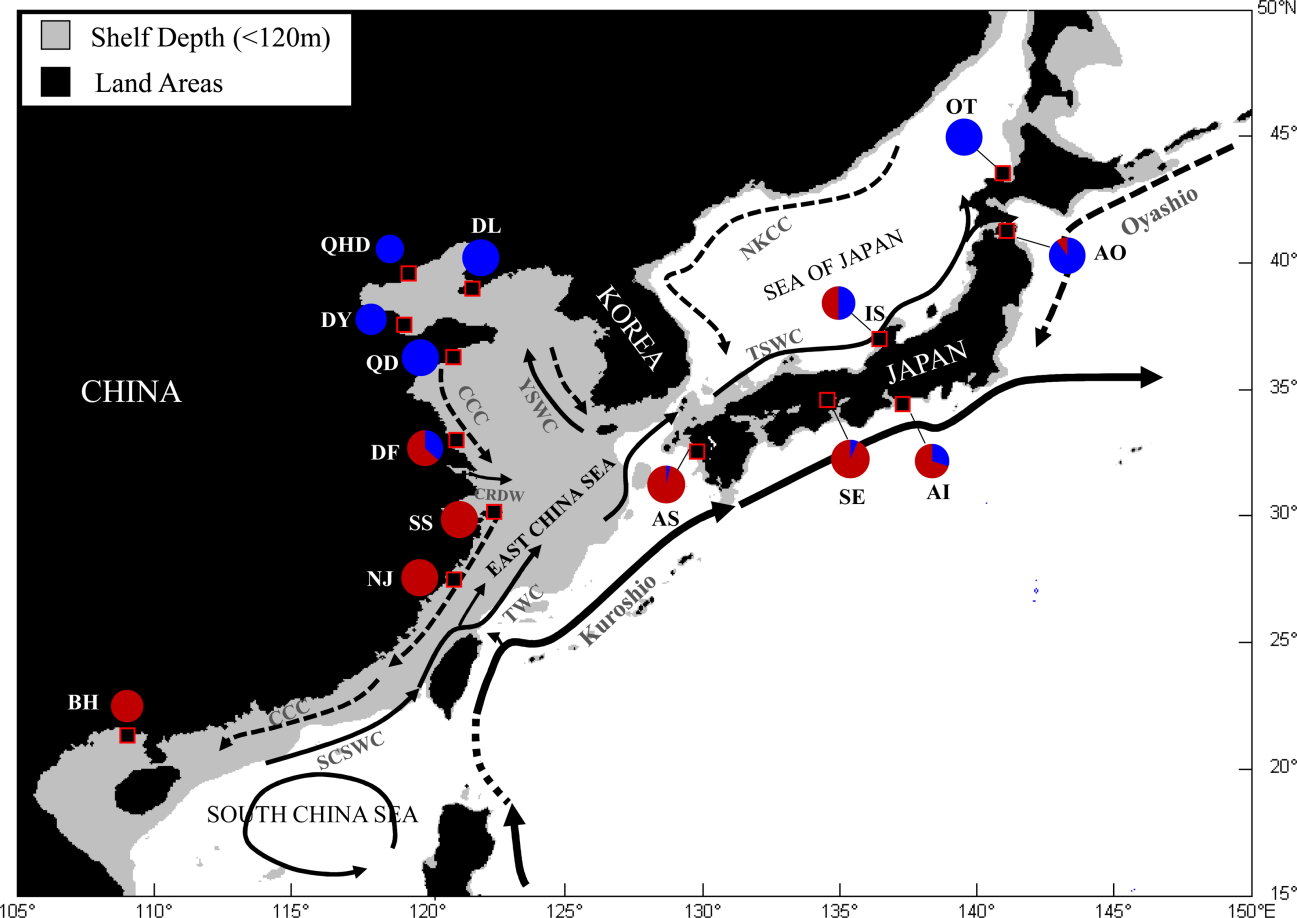
Map showing sample locations of the Japanese mantis shrimp. Pie charts represent the relative frequency of mtDNA COI haplotypes from each site belonging to the two main lineages described in Cheng & Sha (2017). SCSWC, South China Sea Warm Current; TWC, Taiwan Warm Current; CCC, China Coastal Current; CRDW, Changjiang River Diluted Water; YSWC, Yellow Sea Warm Current; TSWC, Tsushima Warm Current; NKCC, North Korea Cold Current. QHD, Qinhuangdao; DL, Dalian; DY, Dongying; QD, Qingdao; DF, Dafeng; SS, Shengshan; NJ, Nanji; BH, Beihai; OT, Otaru; AO, Aomori; IS, Ishikawa; AI, Aichi; SE, Setonaikai; AS, Ariake Sea.

A total of 329 adult *O. oratoria* individuals were collected from 14 localities along the coast of China and Japan during 2013 to 2015 (Table 1, Fig. 1). Samples were collected by using the commercial shrimp trawler in the regions and from local fish markets. In both cases, we confirmed that the mantis shrimps were caught in neighboring coastal waters of the area. The Institutional Animal Care and Use Committee (IACUC) of the Chinese Academy of Sciences provided full approval for this research (No. 2011–2). Specimens were preserved in 95% ethanol and frozen (−20 °C) for DNA extraction. Total genomic DNA was extracted from abdominal muscle using a DNeasy blood and tissue kit (Qiagen, USA) following the manufacturer’s protocol.

**Table 1:** Details of the sampling information and summary statistics^a^ for microsatellite DNA variability in *O. oratoria*.

Location	Abb.	Longitude, latitude	Date of collection	Sample size	*N*_a_	*A*_R_	*H*_o_	*H*_E_	PIC
Qinhuangdao	QHD	119.66°E, 39.91°N	2014.05	24	5.6	5.336	0.613	0.666	0.598
Dalian	DL	121.56°E, 38.87°N	2014.05	24	5.4	5.137	0.529	0.597	0.537
Dongying	DY	119.36°E, 37.50°N	2014.11	23	5.7	5.530	0.600	0.617	0.565
Qingdao	QD	120.34°E, 36.06°N	2014.05	24	5.5	5.277	0.604	0.596	0.543
Otaru	OT	140.98°E, 43.39°N	2015.06	24	6.1	5.854	0.483	0.628	0.575
Aomori	AO	140.74°E, 41.09°N	2015.10	24	5.2	5.035	0.508	0.668	0.596
Ishikawa	IS	136.25°E, 36.80°N	2013.1	20	5.4	5.400	0.465	0.657	0.601
Aichi	AI	137.08°E, 34.48°N	2015.10	22	5.6	5.488	0.436	0.629	0.576
Setonaikai	SE	133.89°E, 34.44°N	2015.10	24	6.1	5.817	0.521	0.637	0.583
Ariake Sea	AS	130.32°E, 32.93°N	2015.02	24	5.7	5.467	0.467	0.617	0.565
Dafeng	DF	121.30°E, 33.44°N	2015.05	24	6.3	6.049	0.504	0.718	0.654
Shengshan	SS	122.50°E, 30.75°N	2015.07	24	6.0	5.803	0.438	0.616	0.565
Nanji	NJ	121.01°E, 27.49°N	2014.06	24	5.8	5.588	0.517	0.636	0.575
Beihai	BH	108.88°E, 21.35°N	2015.07	24	7.1	6.847	0.488	0.721	0.675

**Notes.**

a Summary statistics include mean number of allelic number (*N*_a_), allelic richness (*A*_R_), observed heterozygosity (*H*_O_), expected heterozygosity (*H*_E_) and polymorphism information content (PIC).

### Microsatellite isolation and genotyping

A total of 24 microsatellite loci were developed for *O. oratoria* through transcriptome sequencing (Cheng, Zhang & Sha, 2018). In this study, ten of 24 microsatellites were chosen that were successfully amplified across 14 sampling localities and exhibited polymorphisms. The sequence information of ten microsatellite loci is listed in Table S1. The PCR amplifications followed the PCR protocol reported by Cheng, Zhang & Sha (2018). The PCR products were genotyped on an ABI Prism 3730 DNA Analyzer and were further scored using GeneMapper v4.0 (Applied Biosystems, USA).

### Population genetic analysis

Microsatellite genetic diversity was quantified as number of alleles (*N*_a_), observed heterozygosity (*H*_o_) and expected heterozygosity (*H*_E_) for each locus and population using the Excel Microsatellite Toolkit (Park, 2001). Polymorphism information content (PIC) was calculated using allele frequencies according to the formula given by Botstein et al. (1980). Allelic richness (*A*_R_), which is a standardized index of the mean number of alleles per locus irrespective of sample size, was calculated with Fstat v2.9.3 (Goudet, 2001). Test for deviations from Hardy-Weinberg equilibrium (heterozygote deficits) and linkage disequilibrium was performed with 10,000 burn-in steps, and 500 batches of 5000 Monte Carlo Markov Chain (MCMC) steps per batch as implemented in GENEPOP v4.0.6 (Rousset, 2007). The significance levels were adjusted for multiple simultaneous tests using the sequential Bonferroni correction (Rice, 1989).

Several alternative methods were used to infer genetic structure of *O. oratoria* populations. The estimator *F*_ST_ was used to assess the amount of genetic differentiation among populations according to Weir & Cockerham (1984) using Fstat v2.9.3 (Goudet, 2001) and significance estimated using permutation tests (1,000 replicates). Mantel tests were conducted to determine if there was evidence for a relationship between genetic (Slatkin’s linear *F*_ST_) and geographic (kilometers) distances as implemented in ARLEQUIN v3.5 (Excoffier & Lischer, 2010). Poptree2 (Takezaki, Nei & Tamura, 2010) was used to construct UPGMA dendrogram based on a pairwise *D*_A_ distance (Nei, Tajima & Tateno, 1983) matrix using 1,000 bootstrap replicates. A model-based Bayesian clustering algorithm was undertaken using STRUCTURE v2.3.4 (Pritchard, Stephens & Donnelly, 2000) to determine the most possible number of genetic discrete groups (*K*) and the admixture proportion (*Q*) of the individuals to the inferred group based on their allele frequencies. The simulations were conducted assuming an admixture model with correlated allele frequencies. The simulated *K* value was tested from 1 to 14 with 10 iterations at each level of *K* to verify the consistency of the results. To ensure the discovery of additional cryptical genetic groups, we further preformed STRUCTURE analyses on the six temperate resident populations for cluster models of *K* = 1–6, and the eight populations in the subtropical and tropical region for cluster models of *K* = 1–8, respectively. All analyses were run with a burn-in period of 100,000 steps and 1000,000 MCMC steps after burn-in. The optimal value of *K* that best fits the data was estimated using Evanno’s Δ*K* method (Evanno, Regnaut & Goudet, 2005) as implemented in STRUCTURE SELECTOR (Li & Liu, 2018). Major modes in the STRUCTURE output were identified using CLUMPAK (Kopelman et al., 2015) and visualized in Distruct v1.1 (Rosenberg, 2004).

Discriminant Analysis of Principal Components (DAPC) (Jombart, Devillard & Balloux, 2010) was also applied to examine the genetic relationships among populations by using the “adegenet 2.0.1” package (Jombart, 2008) in R 3.6.2 software (R Development Core Team, 2015). To further determine *O. oratoria* population structure, a hierarchical analysis of molecular variance (AMOVA) was performed with ARLEQUIN v3.5 (Excoffier & Lischer, 2010) to estimate the partitioning of genetic variation within and among putative groupings of samples. In all multiple tests, significance was determined by 1,000 random permutations and *P* values from multiple comparisons were Bonferroni adjusted (Rice, 1989).

### Hybrid identification and introgression analysis

Since two genetic backgrounds were detected in population AO, IS, SS (see Results section), the USEPOPINFO model which uses sampling locations to detect migrants or hybrids was applied to test the hypothesis of mixed ancestry for individuals in AO, IS and SS during the past two generations (i.e., GENSBACK = 2) as implemented in STRUCTURE. Two main clusters were defined according to the results of STRUCTURE, DAPC and UPGMA dendrogram, with one containing individuals from populations in the temperate region and the other containing those collected from the subtropical and tropical region. We ran STRUCTURE for 1000,000 iterations after a burn-in of 100,000 iterations and fixed the number of populations to *K* = 2. Individuals with less than 50% posterior probability (*q*) of having pure ancestry from the assumed population were considered as hybrids or migrants (Pritchard, Stephens & Donnelly, 2000; Falush, Stephens & Pritchard, 2007).

To further investigate the introgression pattern in populations from the sympatric areas, we estimated the hybrid index and interspecific heterozygosity by using the package “INTROGRESS” (Gompert & Alex Buerkle, 2010) in R 3.6.2 software (R Development Core Team, 2015). Based on the results of the STRUCTURE analysis, the temperate resident populations were defined as parental population 1, while those from the subtropical and tropical region were designed as parental population 2. Individuals with admixture proportions *Q* > 0.9 from the STRUCTURE results for either group were used as reference for parental populations in hybrid index calculations. Values of the hybrid index refer to the proportion of alleles derived from parental population 2 for each admixed individual. Values of the interspecific heterozygosity provide the proportion of the individual’s genome with alleles inherited from both parental populations (Buerkle, 2005). The interspecific heterozygosity was then plotted against the hybrid index to show a two-dimensional representation of genomic composition for each admixed individual.

## Results

### Genetic diversity

Genetic polymorphism in terms of average number of alleles, observed and expected heterozygosities varied depending on the locus and population (Table 1, Table S2). The average number of alleles per locus (*N*_a_) over samples ranged from 3.6 for c32844 to 9.6 for c45952, and the average number of alleles per population ranged from 5.2 (AO) to 7.1 (BH). Allelic richness (*A*_R_) varied between 5.035 (AO) to 6.847 (BH). The average of observed heterozygosity (*H*_O_) across loci per population varied from 0.436 (AI) to 0.613 (QHD), and the average of expected heterozygosity (*H*_E_) across loci varied from 0.596 (QD) to 0.721 (BH). The population with the highest value of polymorphism information content was the population BH (0.675), whereas that with the lowest value was the population DL (0.537). Significant departure from Hardy-Weinberg equilibrium was detected for 54 out of the 140 locus-by-population tests after Bonferroni correction. No linkage disequilibrium was detected for populations across loci after Bonferroni correction.

### Population genetic structure

**Figure 2:**
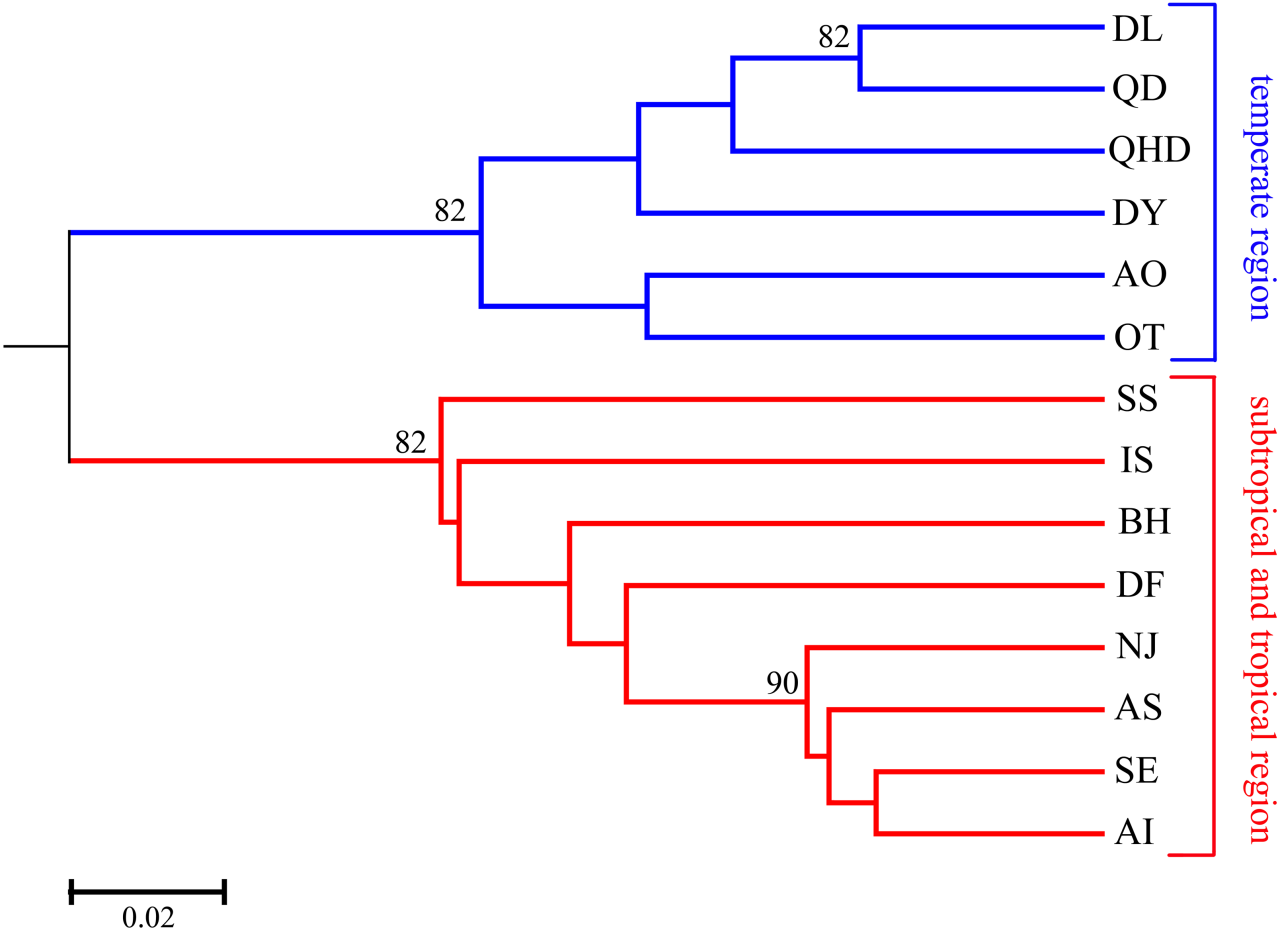
UPGMA tree based on *D*_*A*_ distance among 14 *O. oratoria* populations. Numbers above branches show posterior probabilities of nodes. QHD, Qinhuangdao; DL, Dalian; DY, Dongying; QD, Qingdao; DF, Dafeng; SS, Shengshan; NJ, Nanji; BH, Beihai; OT, Otaru; AO, Aomori; IS, Ishikawa; AI, Aichi; SE, Setonaikai; AS, Ariake Sea.

As illustrated in our previous study (Cheng & Sha, 2017), two distribution regions were defined for the sampled *O. oratoria* populations according to the distribution of mtCOI haplotypes and nrDNA ribotypes: temperate region (including the Bohai and Yellow Seas as well as the northern Japan Sea), and subtropical and tropical region (including the East and South China Seas as well as the southern Pacific coast of Japan). Pairwise *F*_ST_ values between populations of different regions were high (0.081–0.239) and all statistically significant after Bonferroni correction, whereas most of the pairwise *F*_ST_ values between populations within each region was relatively low (Table 2). The UPGMA dendrogram indicated that all *O. oratoria* populations were divided into two distinct clusters according to their geographical origin. Populations from the temperate region showed closer clustering relationships and the other populations from the subtropical and tropical region clustered together (Fig. 2). A significant pattern of differentiation was observed over all populations (*F*_ST_ = 0.149, *P* = 0.000; Table 3). The results of AMOVA also revealed significant genetic differentiation at all hierarchical levels with more genetic variation partitioned by regions (15.73%, *P* = 0.000) than that occurring among populations within regions (5.48%, *P* = 0.000) (Table 3).

**Figure 3:**
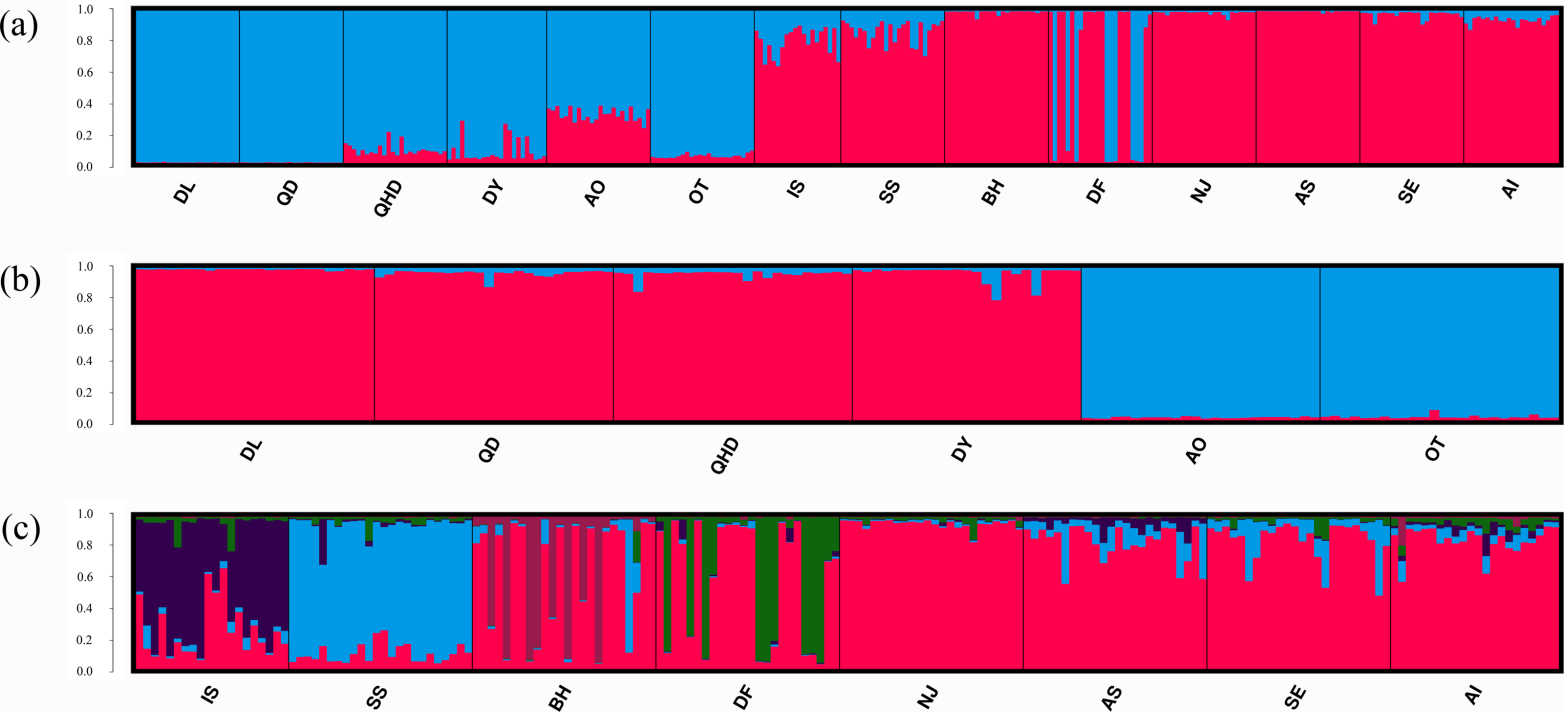
Population structure of *O. oratoria* populations obtained from STRUCTURE analysis. STRUCTURE output with *K* = 2 (A) showing population structure among all 14 populations; subsequent analysis supporting two genetic clusters (*K* = 2) for population in the temperate region (B) and five clusters (*K* = 5) for the rest populations collected from the subtropical and tropical region (C). The horizontal axis indicates individuals grouped by populations and the vertical axis indicates the posterior probability of assignment to the detected clusters. QHD, Qinhuangdao; DL, Dalian; DY, Dongying; QD, Qingdao; DF, Dafeng; SS, Shengshan; NJ, Nanji; BH, Beihai; OT, Otaru; AO, Aomori; IS, Ishikawa; AI, Aichi; SE, Setonaikai; AS, Ariake Sea.

Results of the Bayesian cluster analysis indicated that the presence of two genetic clusters best fitted the microsatellite data (*K* = 2, Fig. 3A, Fig. S1A), mirroring the results of UPGMA dendrogram. One genetic group consisted of most individuals from populations in the temperate region as well as nine individuals in DF from the sympatric zone, and the other genetic group was formed by individuals collected from the subtropical and tropical region as well as the remaining 15 individuals in DF. However, two genetic backgrounds were detected in the population AO, IS and SS, implying their intermediated genotypes and hybrid origins. When considering the two regions separately, fine structure was found in each genetic cluster. For the populations in the temperate region, the Japanese populations (AO and OT) was genetically separated from the four Chinese populations (Fig. 3B, Fig. S1B). For the remaining eight populations from the subtropical and tropical region, the model with *K* = 5 resulted in the highest Δ*K* (Fig. 3C, Fig. S1C). For the population IS, SS, BH, DF, each of them represented one genetic cluster, while individuals from the rest four populations were assigned to the fifth cluster.

**Table 2:** Matrix of pairwise *F*_ST_ values between 14 *O. oratoria* populations based on 10 microsatellite loci.

	DL	QD	QHD	DY	AO	OT	DF	IS	SS	BH	NJ	AS	SE
QD	0.023												
QHD	0.049^*^	0.053^*^											
DY	0.052^*^	0.112^*^	0.051^*^										
AO	0.087^*^	0.103^*^	0.062^*^	0.085^*^									
OT	0.065^*^	0.060^*^	0.091^*^	0.112^*^	0.046^*^								
DF	0.107^*^	0.127^*^	0.070^*^	0.083^*^	0.043^*^	0.121^*^							
IS	0.200^*^	0.181^*^	0.147^*^	0.216^*^	0.081^*^	0.159^*^	0.069^*^						
SS	0.213^*^	0.206^*^	0.184^*^	0.198^*^	0.090^*^	0.157^*^	0.067^*^	0.098^*^					
BH	0.211^*^	0.201^*^	0.149^*^	0.197^*^	0.102^*^	0.175^*^	0.053^*^	0.076^*^	0.070^*^				
NJ	0.230^*^	0.232^*^	0.147^*^	0.199^*^	0.106^*^	0.210^*^	0.040^*^	0.092^*^	0.083^*^	0.033			
AS	0.239^*^	0.227^*^	0.165^*^	0.234^*^	0.114^*^	0.204^*^	0.056^*^	0.068^*^	0.072^*^	0.029	0.013		
SE	0.215^*^	0.195^*^	0.153^*^	0.217^*^	0.093^*^	0.179^*^	0.041	0.069^*^	0.040^*^	0.025	0.017	0.007	
AI	0.218^*^	0.193^*^	0.154^*^	0.229^*^	0.098^*^	0.184^*^	0.055^*^	0.054^*^	0.071^*^	0.026	0.037^*^	0.013	0.009

**Notes.**

QHD Qinhuangdao DL Dalian DY Dongying QD Qingdao DF Dafeng SS Shengshan NJ Nanji BH Beihai OT Otaru AO Aomori IS Ishikawa AI Aichi SE Setonaikai AS Ariake Sea

Pairwise *F*_ST_ values between populations of different regions are highlighted in bold.

* *P* < 0.05.

**Table 3:** Analysis of molecular variance (AMOVA) for different hierarchical analyses of *O. oratoria* populations.

Structure tested	Observed partition				*P*
	df	Variance	% total	*F* Statistics	
*One gene pool*					
Among populations	13	0.428	14.91	*F*_ST_=0.149	**0.000**
Within populations	644	2.441	85.09		
*Two gene pools* (DL, QD, QHD, DY, AO, OT) (IS, SS, BH, DF, NJ, AS, SE, AI)					
Among groups	1	0.487	15.73	*F*_CT_ = 0.157	**0.000**
Among populations/within groups	12	0.170	5.48	*F*_SC_ = 0.065	**0.000**
Within populations	644	2.441	78.79	*F*_ST_ = 0.212	**0.000**

**Notes.**

QHD Qinhuangdao DL Dalian DY Dongying QD Qingdao DF Dafeng SS Shengshan NJ Nanji BH Beihai OT Otaru AO Aomori IS Ishikawa AI Aichi SE Setonaikai AS Ariake Sea

* Bold *P* numbers are significant values.

**Figure 4:**
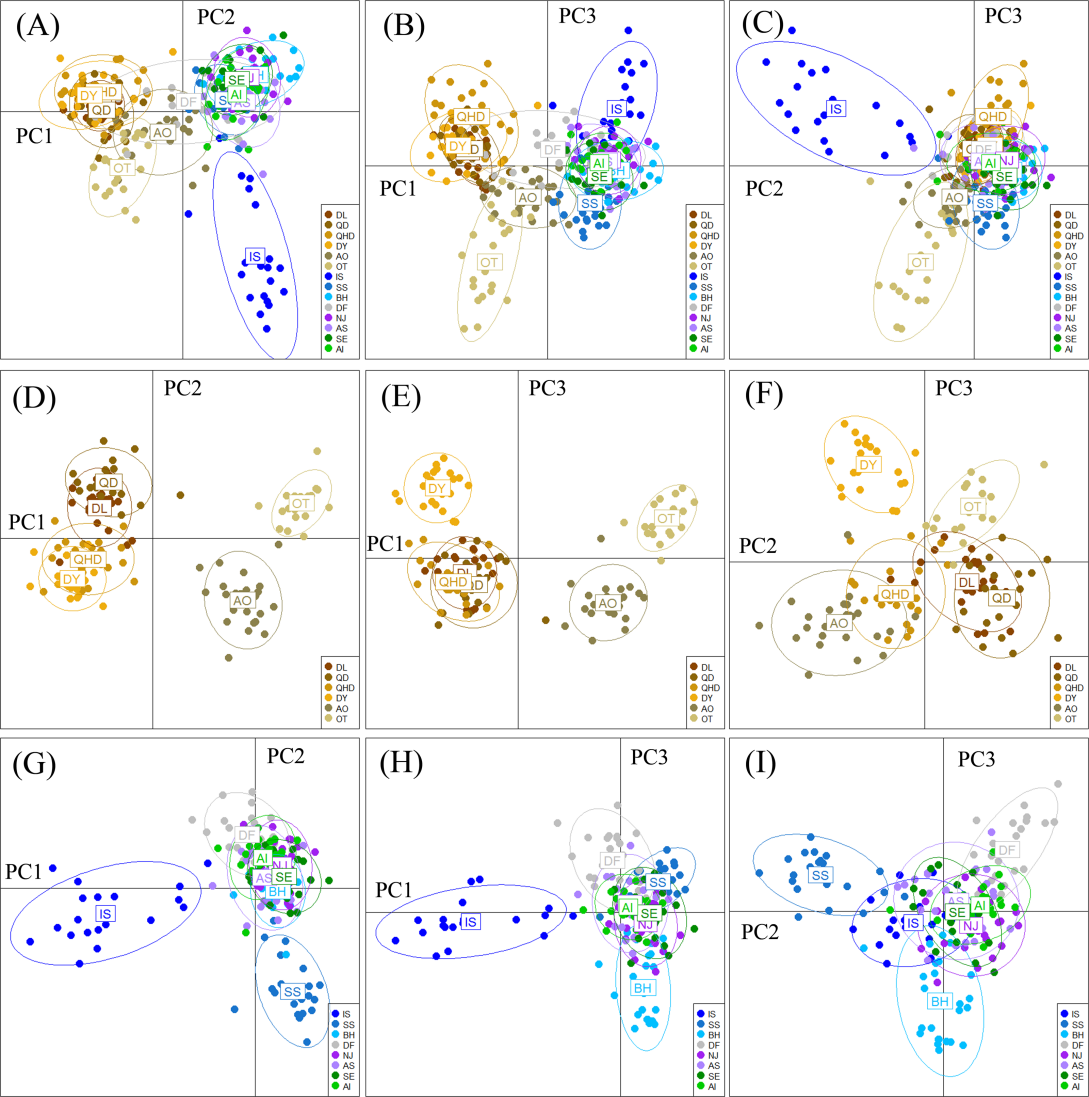
Results of Discriminant Analysis of Principal Components (DAPC) plots using the first three components. Plot of DAPC scatters of all 14 *O. oratoria* populations (A–C), six temperate resident populations (D–F), and the eight populations collected from the subtropical and tropical region (G–I). QHD, Qinhuangdao; DL, Dalian; DY, Dongying; QD, Qingdao; DF, Dafeng; SS, Shengshan; NJ, Nanji; BH, Beihai; OT, Otaru; AO, Aomori; IS, Ishikawa; AI, Aichi; SE, Setonaikai; AS, Ariake Sea.

The DAPC plotting using the first three principle components resolved a similar clustering pattern to that of the STRUCTURE analysis (Fig. 4). For all populations, ellipses for the populations in the temperate region overlapped, and the rest populations in the subtropical and tropical region were grouped tightly, except for the population DF whose ellipse overlapped with those of populations from the two regions (Figs. 4A–4C). For the six temperate resident populations, DAPC plotting indicated individuals from the coastal waters of China were clustered together and formed a separate genetic group from those in the northern Japan Sea (Figs. 4D–4F). For the rest eight populations, DAPC plotting indicated that the population IS and SS showed distinct clustering and ellipses for the rest populations overlapped (Figs. 4G–4I). When considering the Chinese and Japanese populations, respectively, a north–south subdivision was detected in both regions (Fig. S2). Although there was no evidence of positive correlation between genetic and geographic distances when all pairwise comparisons were included (*r* = 0.112, *P* = 0.182), this relationship was not consistent across the range of *O. oratoria* (Fig. S3). The Mantel tests indicated that a significant positive relationship was detected between genetic and geographic distances for pairwise comparisons between populations from the temperate region (*r* = 0.702, *P* = 0.05, Fig. S3), from the China coastal waters (*r* = 0.427, *P* = 0.018, Fig. S3), and from the Japan coastal waters (*r* = 0.64, *P* = 0.002, Fig. S3). However, this relationship was not significant when populations from the subtropical and tropical region were tested (*r* = 0.028, *P* = 0.403, Fig. S3).

### Signature of introgression in *O. oratoria*

**Figure 5:**
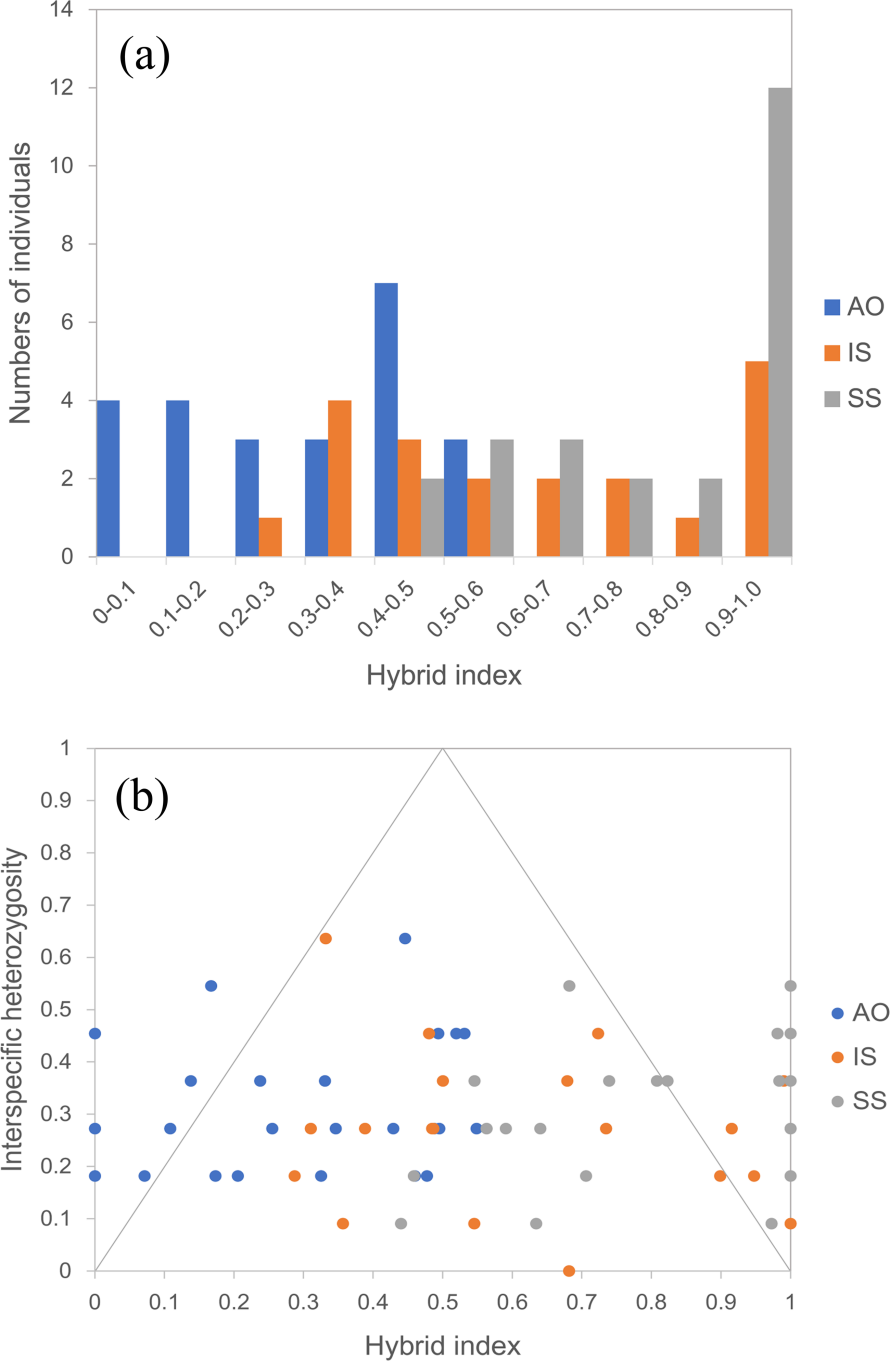
Hybrid index assuming that the temperate resident populations are defined as parental population 1 and those from the subtropical and tropical region are designed as parental population 2. (A) Histogram showing the distribution of hybrid indices of each individual in all three hybrid populations. Hybrid index indicates the fraction of alleles derived from parental population 2, where “0” is pure parental population 1, and “1” is pure parental population 2. (B) Interspecific heterozygosity *vs* hybrid index in three hybrid populations of *O. oratoria*. AO, Aomori; IS, Ishikawa; SS, Shengshan.

By using the USEPOPINFO model in STRUCTURE analysis, 14 individuals in the population AO, 14 individuals in the population IS and 18 individuals in the population SS were identified to have mixed ancestry with high probability (Table S3). The analysis of hybrid index and interspecific heterozygosity estimates also detected extensive hybrid descendants in populations from the sympatric areas. Hybrid individuals collectively showed asymmetry in introgression, as evidenced by a shift of the peal of the histogram (Fig. 5A). Specifically, hybrids in the population AO had greater genetic contribution from the temperate resident group than the group inhabiting the subtropical and tropical region, whereas an opposite trend was observed for hybrids in population IS and SS. The genomic composition of all three hybrid populations showed a broad range of ancestry (hybrid index) and low interspecific heterozygosity (Fig. 5B), indicating a recombinant nature in hybrids.

## Discussion

### Multi-factors shaping population genetic structure of *O. oratoria*

Nuclear microsatellite patterns of genetic subdivision provided further evidence for cryptic speciation in *O. oratoria*. On basis of microsatellite data, we found that a series of analyses, including pairwise *F*_ST_ statistics, STRUCTURE analysis, DAPC clustering, AMOVA analysis and UPGMA phylogenetic tree, all revealed two significantly divergent clades in the Japanese mantis shrimp, even though hybridization occurred in their sympatric zones. The two genetically different clades were geographically structured, with one dominating the temperate zone of the NW Pacific and the other occurring in the subtropical and tropical waters where are influenced by the Kuroshio Current. We dated that the two clades (or lineages) had diverged during the mid-Pliocene (c. 3.85 Mya; Cheng & Sha, 2017). The isolation of the Japan Sea and the South China Sea during glacial periods at mid-Pliocene might serve as vicariant barriers and promote diversification in *O. oratoria*. Further evidence for allopatric differentiation is that geographically distant populations in the same clade have larger genetic similarity than closer populations belonging to different clades as revealed by both mitochondrial DNA and nuclear microsatellites. The duration of the glacial period was much longer than that of the interglacial period, providing enough time for accumulating differentiation to diversification and speciation (Xu & Chu, 2012).

Besides historical events, the pattern of genetic differentiation in *O. oratoria* was also influenced by contemporary factors. Our microsatellite study detected a marked genetic break among China coastal populations, which is line with the Changjiang Estuary. A recent population genetic study based on mitochondrial 16S rRNA also arrived at a similar conclusion that the Changjiang River outflow might act as an oceanic barrier to gene flow in *O. oratoria* (Du et al., 2016). As the third largest river in the world, the Changjiang River has an average annual discharge of 8∼9 × 10^11^ m^3^ (Fang et al., 2011). The huge freshwater outflow named Changjiang River Diluted Water (CRDW) can dramatically influence surrounding ocean currents (Beardsley et al., 1985) and salinity of the upper layer of the Kuroshio Current (Wu, Chen & Liu, 2010), which was assumed to have played an important role in driving the biogeographic pattern of coastal species communities (Wang, Tsang & Dong, 2015). Similar to present study, significant genetic divergence in line with the Changjiang River outflow has been reported in some other marine species, such as the bivalve *Cyclina sinensis* (Zhao et al., 2009), macroalga *Sargassum hemiphyllum* (Cheang, Chu & Ang, 2010), gastropod *Cellana toreuma* (Dong et al., 2012), and cocktail shrimp *Trachypenaeus curvirostris* (Han et al., 2015). However, the barrier effect of the Changjiang River outflow on gene flow of *O. oratoria* populations might have weakened as consequence of the coupled impacts of climate change and human activities (Wang, Tsang & Dong, 2015), which resulted in a sympatric zone in the southern Yellow Sea and caused the occurrence of intermediated genotypes in the north of East China Sea.

In the case of *O. oratoria* populations off Japan coast, genetic discontinuity was also revealed between northern and southern samples. The haplotype frequency of mtDNA lineages in both sides of Japanese Island (Cheng & Sha, 2017) and microsatellite variation between northern and southern groups observed in this study both reflect a candidate sea temperature barrier to gene flow. Surface sea temperature in coastal waters of Japanese Island is mainly influenced by the subtropical warm and saline Kuroshio Current and the subarctic cold and less saline Oyashio Current (Fig. 1). These two currents meet each other off the east coast of the Japanese Island and produce the mixed water mass region, where pronounced environmental gradients (temperature and salinity) are observed in the North Pacific (Oba et al., 2006). Similar latitudinal gradients of sea surface temperature exist in China coastal waters, which attributes to the pattern of oceanic circulation, including cold China Costal Current, warm Kuroshio Current and its branches (Fig. 1). These sharp temperate gradients have been reported to limit the dispersal of marine larvae, as evidenced by the distribution patterns of larval myctophid fishes in the transition zone between the Kuroshio and Oyashio Fronts (Chiyuki et al., 2004). All these findings suggested a crucial role of sea surface temperature in determining the population structure of the Japanese mantis shrimp. Such strong correlations between geographical distribution of genetic variation and seawater temperature differences are also evident in other marine species in the NW Pacific, such as the flathead mullet *Mugil cephalus* (Shen et al., 2011) and the Japanese sand lance *Ammodytes personatus* (Han et al., 2012).

Nuclear microsatellite data can provide different genetic information about population structure compared with sequence data from either nuclear or mitochondrial genome. Although high rates of mutation and homoplasy make microsatellites more likely to obscure population history (Zink & Barrowclough, 2008), the high mutation rates of microsatellites may result in greater statistical power in uncovering population structure that was not apparent using less variable markers (Hedrick, 1999; Nielsen et al., 2001). In this study, our microsatellite data indicated significant genetic differentiation between Chinese and Japanese populations inhabiting temperate region, which was not detected in our previous analysis of mitochondrial and nuclear DNA sequences (Cheng & Sha, 2017). Additionally, we found that genetic differentiation among temperate resident populations was strongly predicted by geographic distance (*r* = 0.702, *P* = 0.05), suggesting the presence of isolation by distance. However, no significant correlation between genetic and geographic distances was observed for populations from the subtropical and tropical region (*r* = 0.028, *P* = 0.403). This pattern is not unexpected considering the oceanographic current systems of this region. The NW Pacific is dominated by the Kuroshio Current (Fig. 1), a movement of 30–50 million m^3^ s^−1^ of waters from the east of the Philippine Islands, with the main branch flowing north-east along the eastern coast of Taiwan towards the southern coast of Kyushu. The minor branches further flow northward as shallow surface currents, such as Yellow Sea Warm Current (YSWC) and Tsushima Warm Current (TSWC) (Hsueh, Wang & Chern, 1992). At the mercy of these currents, pelagic larvae of *O. oratoria* from the South China Sea could follow the northward current to the East China Sea and to the coast of Japan, consequently facilitating gene flow and genetic cohesion throughout this region. This finding is not unusual because the Kuroshio Current is reported to have facilitated a great number of warm-water marine species migrate northward from their tropical center and expand their distribution ranges (Liu, 2013).

### Hybridization in the sympatric region

As a source of evolutionary innovation, hybridization may provide an opportunity for the formation of new hybrid taxa and subsequent introgression may promote adaptive divergence potentially resulting in speciation (Abbott et al., 2013). Contrary to the extensive information on hybridization in terrestrial and freshwater organisms, studies focusing on the marine realm are still scare (Arnold & Fogarty, 2009), which also holds for marine invertebrates. In this study, extensive introgression was observed in the three sympatric populations of *O. oratoria* (AO, IS, SS), where the majority of individuals in the hybrid zones were characterized with broad ancestry levels and low interspecific heterozygosity, indicative of recombinant hybrid generations. The results are coincident with our recent study that the recombinant nrDNA ITS sequence and intra-individual ITS polymorphism demonstrated the occurrence of hybridization in *O. oratoria* (Cheng & Sha, 2017). It should be noted that intermediated genotypes could have resulted from the retention of ancestral polymorphisms and incomplete lineage sorting. However, the extensive introgression observed in both China and Japan coastal waters makes ancestral polymorphisms unlikely to explain the pattern observed. Given the clear allopatric differentiation (e.g., STRUCTURE results), secondary contact after a long-time isolation seems to be a more plausible explanation. The repeated range shifts associated with glacial climate oscillations might have induced repeated contacts between these two geographically isolated *O. oratoria* lineages, thereby increasing the chance for hybridization, as previously postulated by other studies in the contact zones of the NW Pacific (Xu & Chu, 2012; Ding et al., 2018).

Increasing evidence has revealed northward distribution range shifts in a variety range of marine taxa, with the poleward retreat of temperate species and northern expansion of tropical species (Parmesan & Yohe, 2003; Berge et al., 2005; Parmesan, 2006). Such changes in distribution are likely due to elevated sea surface temperature and shifts in the oceanographic regime (Stenseth et al., 2002; Helmuth et al., 2006; Tsang et al., 2013). In the marine realm, species dispersal range and community structure are highly sensitive to variation in oceanic current patterns, even minor perturbation (Field et al., 2006; Barth et al., 2007). The influence of climate warming on the invertebrate communities has been reported in rocky intertidal areas of California (Barry et al., 1995). Despite less knowledge about community response to climate change in the NW Pacific, a northward range shift in *O. oratoria* driven by the increase in oceanic temperature and variation in local current would be plausible. In the coastal waters of China, individuals from the subtropical and tropical region might disperse northward across the mouth of the Changjiang River as the frontal zone between the Kuroshio Current and the Changjiang River outflow shifts northwards according to the strength of the Kuroshio Current (Yang et al., 2012; Yang et al., 2013). Unlike other sympatric areas, no hybrids were detected in the southern Yellow Sea (population DF), so did results of nrDNA ITS dataset (Cheng & Sha, 2017). It is possible that these individuals are relatively recent migrants rather than representatives of a long-standing population, and so there is insufficient time for hybridization to complete.

Our results pointed towards asymmetry in introgression in the hybrid zones. While hybridization and backcross could occur in both directions, the introgression in the population AO was asymmetric towards the temperate resident group and individuals from the population IS and SS have hybrid ancestry favoring the group inhabiting the subtropical and tropical region. Introgressive hybridization is occasionally asymmetric, which is most easily explained by differences in population abundance (Anderson, 1948; Rieseberg, 1997). Asymmetric introgression may be more frequent from a more abundant species to a rare species than vice versa, and hybrids likely have more opportunities to mate with the most common parent at the local scale (Haygood, Ives & Andow, 2003). Asymmetric introgression associated with population density is better known in plants (e.g., Burgess et al., 2005; Lepais et al., 2009; De La Torre, Ingvarsson & Aitken, 2015), and also has been reported in animals, such as avian (Taylor et al., 2014) and salamander (Johnson et al., 2015). An alternative explanation is that offspring from hybrids that are backcross to either parents exhibit reduced fitness in that parental species habitats. Different alleles may be favored in different environments, and these advantageous alleles can be maintained under selection even when there is high gene flow (Barton & Gale, 1993; Hancock et al., 2011). Hybridization may allow the spread of advantageous alleles from one side to another, however, alleles that are adaptive in one environment context could be maladaptive in the other (Barton & Gale, 1993; Abbott et al., 2013), which may lead to reduced fitness of hybrids in particular environments. However, further evidence is wanting and a deeper investigation of genomic clines and hybrid fitness is necessary to better understand the causes of this complex pattern of introgression in the studied hybrid zones, which would provide insights into the role of hybridization in helping marine organisms to respond to the changing climate.

## Conclusions

Our microsatellite results add to the evidence that *O. oratoria* is a species complex consisting of two morphological cryptic but genetically distinct species between which hybridization occurs. The interaction between historical climate shifts and contemporary factors (e.g., freshwater discharge, temperature gradient and isolation by distance) may account for the patterns of genetic differentiation between and within *O. oratoria* cryptic species. Specifically, range fragment associated with glaciation allows accumulation of mutations and subsequently gives rise to allopatric differentiation of *O. oratoria*. The contemporary oceanographic barriers prevent gene flow between the southern and northern group in *O. oratoria* and facilitate the maintenance of the historical patterns. In addition, rang shifts induced by climate changes and oceanographic factors likely lead to contacts between the two *O. oratoria* cryptic species, thereby increasing the chance for hybridization. These results can provide insights into the mechanisms in determining the distribution and genetic structure of coastal species and the biogeography of the NW Pacific, which is important for understanding how marine species evolve in response to rapidly changing environments.

##  Supplemental Information

10.7717/peerj.10270/supp-1 Figure S1 Number of clusters (K) of *O. oratoria* from Bayesian inference (A) all 14 populations, (B) the six temperate resident populations and (C) the eight populations from the subtropical and tropical waters.

10.7717/peerj.10270/supp-2 Figure S2 Results of Discriminant Analysis of Principal Components (DAPC) plots using the first three components considering the Chinese (A) and Japanese (B) *O. oratoria* populations, respectively In the case of China coastal populations, the population DF from the sympatric zone was excluded from the analysis.

10.7717/peerj.10270/supp-3 Figure S3 Graph illustrating the relationship between genetic and geographic distances for each pair of sampling localities (*r*= 0.112, *P*= 0.182) This relationship is also not significant when pairwise comparisons were conducted between populations from the subtropical and tropical region (red color, *r* = 0.028, *P* = 0.403), but significant for pairs between populations from the temperate region (blue color, *r* = 0.702, *P* = 0.05), from the China coastal waters (squares, *r* = 0.427, *P* = 0.018), and from the Japan coastal waters (triangles, *r* = 0.64, *P* = 0.002). Circle dots indicated pairwise comparisons between populations within regions.

10.7717/peerj.10270/supp-4 Table S1 The sequence information of the ten microsatellite primers used in this study

10.7717/peerj.10270/supp-5 Table S2 Summary statistics for microsatellite DNA variability in *O. oratoria* populations

10.7717/peerj.10270/supp-6 Table S3 Ancestry inference for *O. oratoria* individuals in population AO, IS and SS inferred by STRUCTURE Only individuals with less than 50% posterior probability (*q*) of having pure ancestry from the assumed population are shown, which can be considered as migrants or hybrids descendants.

10.7717/peerj.10270/supp-7 File S1 The raw data showing genotypes scored with GeneMapper for 10 microsatellite loci across 329 *O. oratoria* individuals from 14 populations

## Additional Information and Declarations

### Data Availability

The following information was supplied regarding data availability: The raw data are available in the Supplementary File.
