# Supplementary figures and images for: Nuclear microsatellites reveal population genetic structuring and fine-scale pattern of hybridization in the Japanese mantis shrimp Oratosquilla oratoria
Source: PeerJ. 2020 Nov 5;8:e10270. doi: 10.7717/peerj.10270 (PMC7649012; doi:10.7717/peerj.10270)

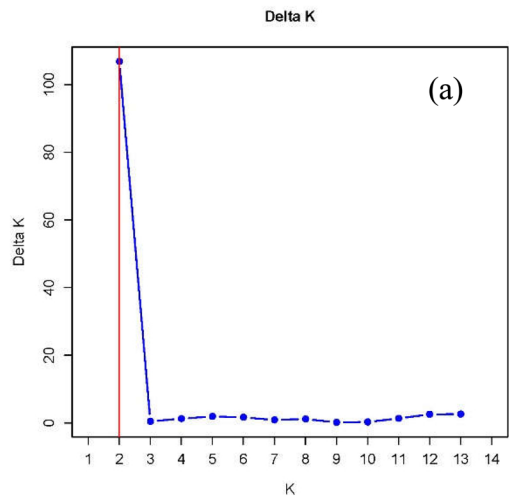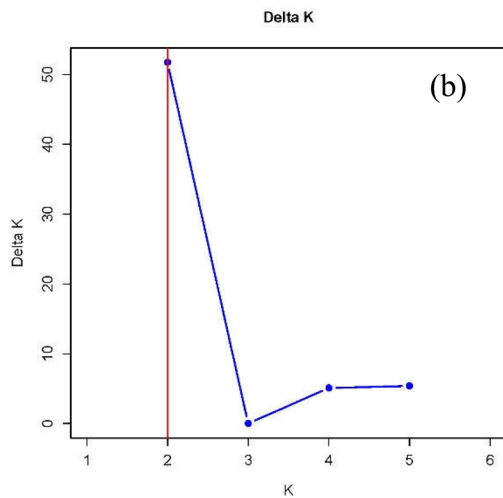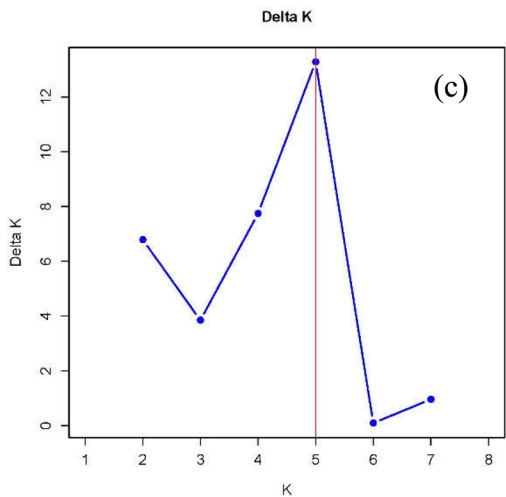

Supplement: Figure S1 — (A) all 14 populations, (B) the six temperate resident populations and (C) the eight populations from the subtropical and tropical waters. [file peerj-08-10270-s001.pdf]

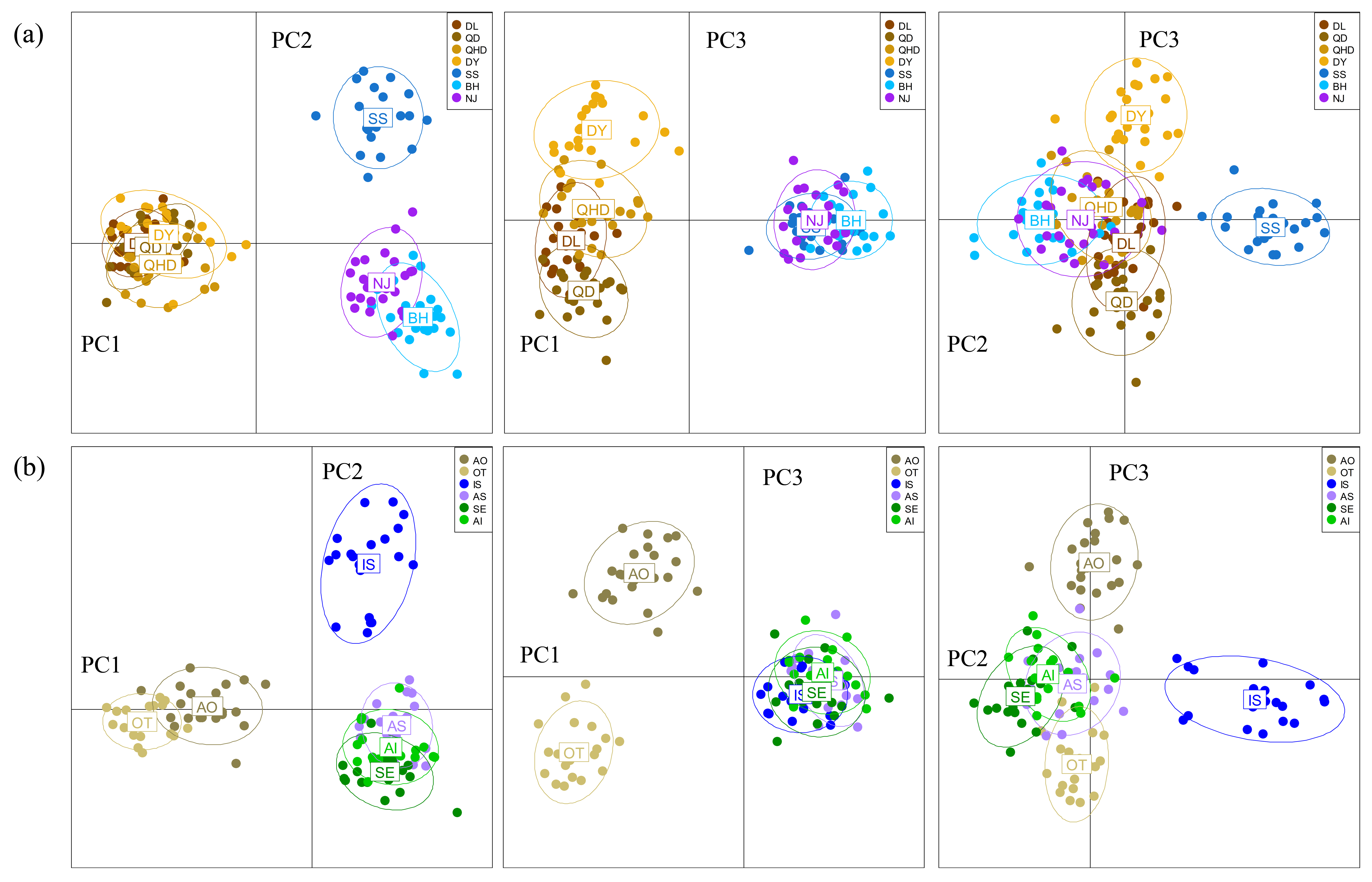

Supplement: Figure S2 — In the case of China coastal populations, the population DF from the sympatric zone was excluded from the analysis. [file peerj-08-10270-s002.png]

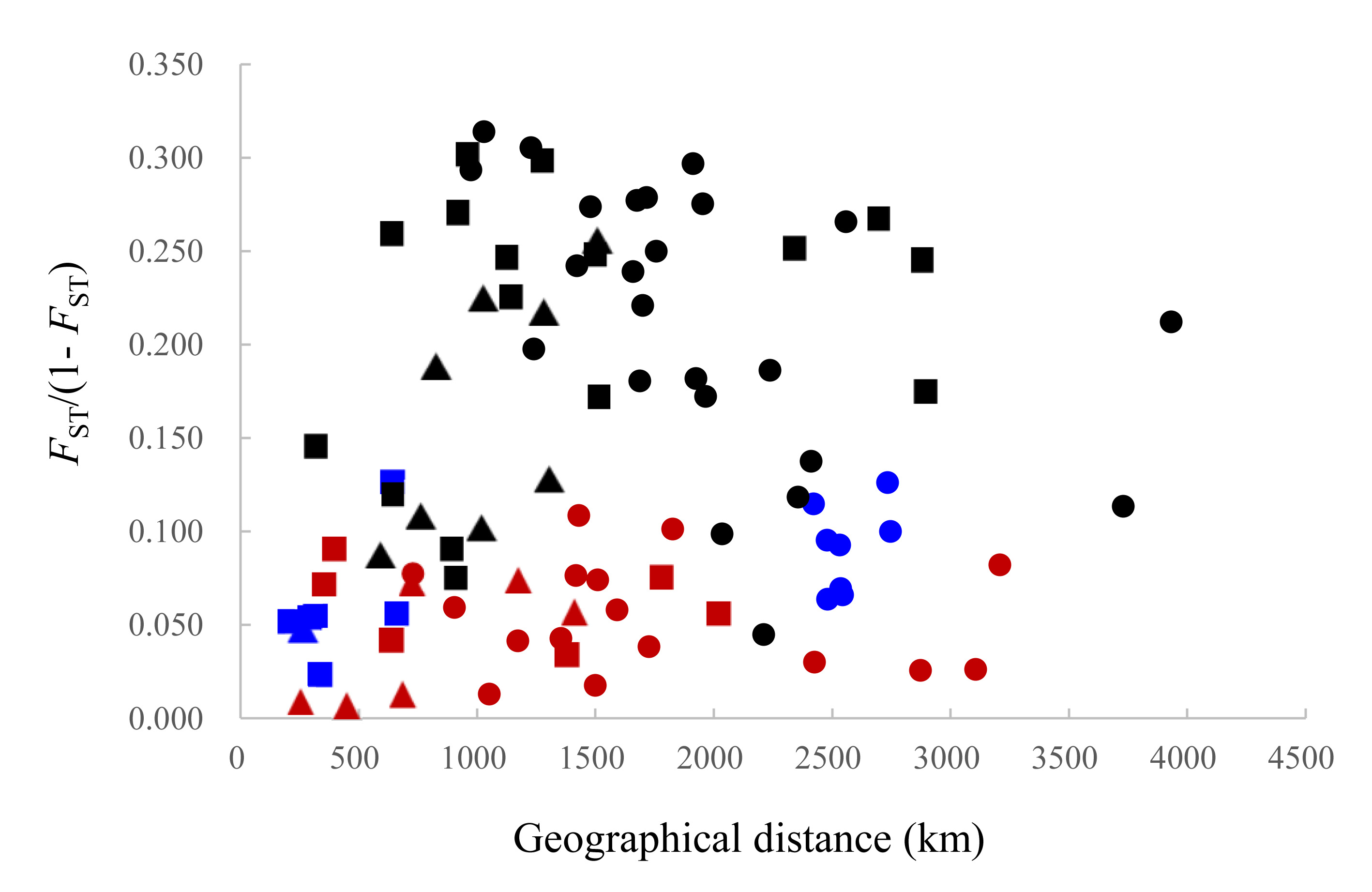

Supplement: Figure S3 — This relationship is also not significant when pairwise comparisons were conducted between populations from the subtropical and tropical region (red color, r = 0.028, P = 0.403), but significant for pairs between populations from the temperate region (blue color, r = 0.702, P = 0.05), from the China coastal waters (squares, r = 0.427, P = 0.018), and from the Japan coastal waters (triangles, r = 0.64, P = 0.002). Circle dots indicated pairwise comparisons between populations within regions. [file peerj-08-10270-s003.png]
